# Lactate-to-albumin ratio index correlates with the occurrence and prognosis of acute kidney injury complicated by cardiac surgery

**DOI:** 10.1016/j.ijcha.2025.101734

**Published:** 2025-07-01

**Authors:** Congying Wang, Xin Sun, Kun Chen, Yaqing Shi, Lili Wang, Shuxia Chen, Dehua Li, Jian Gu

**Affiliations:** a Hebei General Hospital, Shijiazhuang, Hebei, China; b Hebei North University, Zhangjiakou, Hebei, China; c First Affiliated Hospital of Guangzhou Medical University, Guangzhou, China; d DecisionLinnc Dev Group, Hangzhou, China; e Hebei Medical University, Shijiazhuang, Hebei, China

**Keywords:** Lactate-to-albumin ratio, Acute kidney injury, Cardiac surgery, Mortality

## Abstract

**Background:**

The lactate-to-albumin ratio (LAR) is a novel biomarker associated with poor prognosis in critical illnesses. However, its relationship with cardiac surgery associated acute kidney injury (AKI) and prognosis remains unclear. This study aimed to investigate this relationship using data from the MIMIC database.

**Methods:**

A retrospective cohort study was conducted on 11,624 adult cardiac surgery patients admitted to the ICU. Logistic regression, restricted cubic spline (RCS) analysis, and subgroup analysis were used to assess the predictive value of LAR for AKI occurrence and adverse outcomes.

**Results:**

Of the 11,624 patients, 5,965 developed AKI. While LAR concentrations were higher in AKI patients, this association did not persist after multivariate adjustment for potential confounder. Logistic regression showed that LAR was independently associated with in-hospital and ICU mortality, even after adjusting for potential confounders. RCS analysis revealed a non-linear relationship between elevated LAR and increased mortality risk in AKI patients. Correlation analyses demonstrated that LAR was associated with longer hospital and ICU stays and higher SOFA scores. Subgroup analyses consistently showed that elevated LAR was associated with increased mortality risk in older adults, females, and patients with or without hypertension, chronic kidney disease, diabetes, or chronic heart failure.

**Conclusions:**

Among patients with acute kidney injury related to cardiac surgery, especially in those undergoing valve surgery, elevated LAR levels are associated with an increased risk of death, but not with the occurrence of acute kidney injury. Further validation is needed to confirm its predictive role.

## 1 Introduction

Acute kidney injury (AKI) is a common complication following cardiac surgery and strongly impacts patient outcomes. It is reported that the incidence of AKI can reach as high as 40 %, exacerbating the risk of hospital-acquired infections, prolonged hospital stays, and in-hospital mortality [1].The primary cause is acute kidney failure resulting from inadequate renal perfusion, which can lead to multi-organ failure and increased mortality [2]. Since significant clinical manifestations often appear several days after kidney injury, and there is a lack of specific treatment options, early therapeutic interventions and renal protective measures are limited. Therefore, accurate and early identification of risk factors for AKI is crucial. The lactate/albumin ratio (LAR), an emerging biomarker for predicting adverse outcomes, has been shown to have significant prognostic value in diseases such as acute myocardial infarction [3], cardiac arrest [4], sepsis [5], and septic shock [6]. Elevated LAR is associated with increased lactate levels and decreased serum albumin levels. In patients with AKI, elevated lactate levels often indicate tissue hypoperfusion and inadequate oxygenation, while hypoalbuminemia may suggest malnutrition or chronic inflammation—both of which are linked to poor outcomes in AKI patients [7,8]. Therefore, the lactate/albumin ratio holds potential as a key biomarker for assessing the occurrence of AKI and predicting adverse outcomes following cardiac surgery. We conducted a retrospective study to explore the association between LAR and the risk of AKI and its adverse outcomes in patients undergoing cardiac surgery.

## 2 Methods

### 2.1 Data source

This study utilized data from MIMIC-IV version 2.2, an electronic health record dataset of over 50,000 ICU patients admitted to Beth Israel Deaconess Medical Centre (BIDMC) in Boston from 2008 to 2019. The BIDMC Institutional Review Board approved the sharing of research data and waived informed consent. Author DHL accessed the database under certificate number 63768415. Following STROBE guidelines [9].

Inclusion Criteria:

1. Age >18 years and first ICU admission.
2. Undergoing cardiac surgery, including coronary artery bypass grafting (CABG), aortic surgery, valve repair or replacement (single or multiple), or combined procedures.

Exclusion Criteria:

1. ICU stay of less than 24 h.
2. Missing lactate or albumin data upon admission.
3. Missing data >20 %.
4. Patients diagnosed with end-stage renal disease (ESRD).

For the diagnosis of AKI, we followed the criteria set by the 2012 Kidney Disease: Improving Global Outcomes (KDIGO) guidelines [10]. The criteria include: (1) an increase in serum creatinine (SCr) of ≥0.3 mg/dl within 48 h; (2) an increase in SCr to ≥1.5 times the baseline level within 7 days of ICU admission; and (3) urine output less than 0.5 mL/kg/h for 6 consecutive hours. AKI and cardiac surgery data were extracted from the MIMIC-IV database using International Classification of Diseases (ICD) codes (ICD-9 or ICD-10).The baseline creatinine level was the value measured upon admission to the ICU.

Outcomes:

The primary outcomes assessed were in-hospital and ICU mortality rates. Secondary outcomes included the length of hospital stay (LOS-H) and the length of ICU stay (LOS-ICU).

### 2.2 Data extraction

The extracted data are detailed below: (a) Demographic Data: Includes age, gender, heart rate (HR), respiratory rate (RR), systolic blood pressure (SBP), diastolic blood pressure (DBP), mean arterial pressure (MAP), and peripheral oxygen saturation (SpO2).(b)Comorbidities: Comprises conditions such as hypertension, type 2 diabetes mellitus (T2DM), chronic heart failure (CHF), chronic obstructive pulmonary disease (COPD), ischemic heart disease (IHD), chronic kidney disease (CKD), and stroke.(c)Laboratory Data: Encompasses white blood cell count (WBC), platelet count (PLT), hemoglobin (Hb), aspartate aminotransferase (AST), alanine aminotransferase (ALT), blood urea nitrogen (BUN), creatinine, lactate (LAC), fasting blood glucose (FBG), glycosylated hemoglobin (HbA1C), sodium, potassium, and anion gap (AG).(d)Severity of Illness: Measured using the Sequential Organ Failure Assessment (SOFA) score. The lactate-to-albumin ratio (LAR) is defined as follows: LAR = (admission lactate (mmol/L) / albumin (g/dl)).

### 2.3 Statistical analysis

Continuous variables are presented as medians with interquartile ranges, and the Mann-Whitney *U* test was used to compare differences between groups. Categorical variables are expressed as counts and percentages, with chi-square and Fisher’s exact tests employed for intergroup comparisons. Missing values were addressed using multiple imputation methods, while data with missing values exceeding 20 % were excluded. Statistical significance was defined as a two-tailed P-value <0.05. For logistic analysis, both univariate and multivariate models were established to assess the predictive role of the lactate-to-albumin ratio (LAR) on the occurrence of AKI and adverse outcomes following cardiac surgery. Restricted cubic spline (RCS) analysis was utilized to identify potential linear associations between LAR levels and mortality in patients with AKI post-surgery, stratifying the sample based on the optimal threshold. Correlation analysis assessed the relationship between LAR and continuous indicators, including length of stay in hospital (LOS-H), length of stay in ICU (LOS-ICU), and SOFA scores. Additionally, subgroup analyses were conducted based on sex, age, and comorbidities to evaluate potential differences in the incidence of adverse outcomes among AKI patients during ICU and hospital stays. (All Statistical analyses were performed using R version4.2.3 and python version 3.11.4.).

## 3 Results

### 3.1 Association between elevated LAR levels and AKI risk after cardiac surgery

**Fig. 1:**
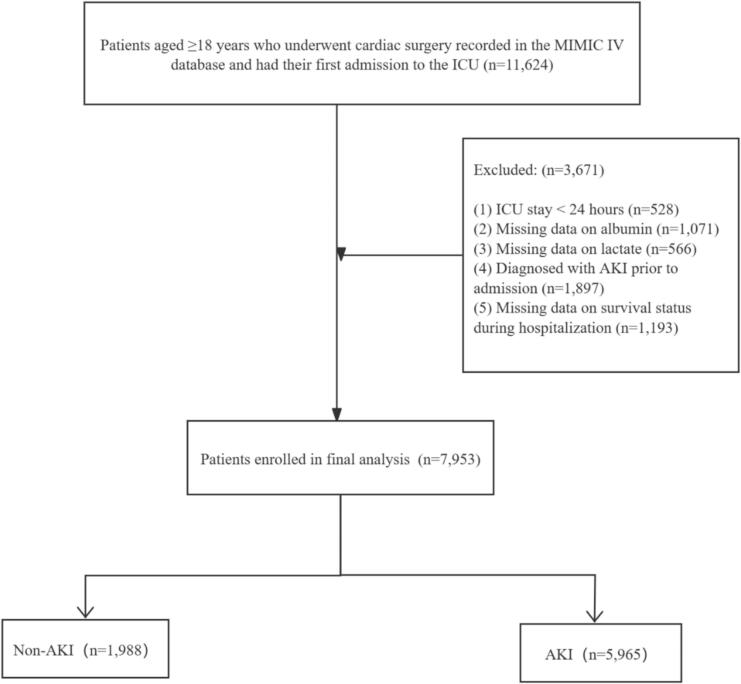
Flow diagram of patient selection from MIMIC-IV databases. AKI, acute kidney injury.

We extracted data on 11,624 patients who underwent cardiac surgery from the MIMIC-IV database (Fig. 1). Variance inflation factor (VIF) values are displayed in Table S1, confirming the absence of multicollinearity between variables. Among these patients, 5,965 developed AKI. As shown in Table 1, there were some differences in baseline characteristics between those with and without AKI post-surgery.

**Table 1:** Baseline characteristics between patients after cardiac surgery with and without AKI.

Characteristics	All surgical patients (n = 7953)	surgical patients without AKI(n = 1988)	surgical patients with AKI(n = 5965)	P valve
Demographic data				
Age, y	68.0(61.0–76.0)	66.0(58.0–73.0)	69.0(61.0–77.0)	＜0.001
Gender, n(%)				0.024
Female	2298 (28.9)	535(26.9)	1763(29.6)	
Male	5655 (71.1)	1453(73.1)	4202(70.4)	
SBP, mmHg	112.0(102.0–123.0)	112.0(103.0–123.0)	112.0(102.0–124.0)	0.874
DBP, mmHg	59.0(52.0–66.0)	59.0(53.0–66.0)	58.0(52.0–65.0)	＜0.001
MAP, mmHg	77.0(70.0–85.0)	78.0(71.0–85.0)	77.0(69.0–85.0)	0.002
HR, bpm	80.0(74.0–86.0)	80.0(73.0–86.0)	80.0(74.0–86.0)	0.338
RR, bpm	15.0(12.0–17.0)	14.0(12.0–17.0)	15.0(12.0–17.0)	0.136
SpO2, %	100.0(99.0–100.0)	100.0(100.0–100.0)	100.0(99.0–100.0)	＜0.001
SOFA	5.0(3.0–7.0)	4.0(3.0–6.0)	5.0(3.0–7.0)	＜0.001

Comorbidities, n(%)				
Hypertension	4822(60.6)	1199(60.3)	3623(60.7)	0.736
T2DM	2506(31.5)	536(26.9)	1970(33.0)	＜0.001
CHF	1831(23.0)	362(18.2)	1469(24.6)	＜0.001
Stroke	595(7.5)	132(6.6)	463(7.8)	0.100
COPD	781(9.8)	168(8.5)	613(10.3)	0.018
CKD	894(11.2)	166(8.4)	728(12.2)	＜0.001
IHD	5955(74.9)	1469(73.9)	4486(75.2)	0.243

Laboratory data				
WBC, M/mcl	7.1(5.8–8.7)	7.0(5.8–8.6)	7.1(5.8–8.7)	0.148
PLT, K/mcl	142.0(114.0–177.0)	141.0(115.0–176.0)	142.0(113.0–177.0)	0.928
Hb, g/dL	9.5(8.4–10.8)	9.7(8.6–10.8)	9.5(8.3–10.7)	＜0.001
AST	22.0(18.0–29.0)	22.0(18.0–28.0)	22.0(18.0–29.0)	0.687
ALT	20.0(15.0–28.0)	21.0(15.0–28.0)	20.0(14.0–28.0)	0.002
TBIL	0.5(0.4–0.7)	0.5(0.3–0.7)	0.5(0.4–0.7)	0.363
LAC	1.3(1.0–1.7)	1.3(1.0–1.7)	1.3(1.0–1.7)	0.543
ALB	4.0(3.6–4.3)	4.1(3.7–4.4)	3.9(3.5–4.3)	＜0.001
LAR	0.3(0.2–0.4)	0.3(0.2–0.4)	0.3(0.3–0.4)	＜0.001
FBG	100.0(90.0–115.0)	98.0(89.0–112.0)	101.0(90.0–115.0)	＜0.001
HbA1c	5.8(5.5–6.5)	5.7(5.4–6.3)	5.8(5.5–6.5)	＜0.001
BUN	14.0(12.0–18.0)	14.0(11.0–17.0)	15.0(12.0–18.0)	＜0.001
Creatinine	0.8(0.7–1.0)	0.8(0.7–0.9)	0.8(0.7–1.0)	＜0.001
Sodium	138.0(136.0–139.0)	137.0(136.0–139.0)	138.0(136.0–139.0)	0.043
Potassium	4.0(3.7–4.2)	4.0(3.7–4.2)	4.0(3.7–4.2)	＜0.001
Calcium	8.3(8.0–8.6)	8.3(8.0–8.7)	8.3(8.0–8.6)	0.277
AG	11.0(9.0–12.0)	10.0(9.0–12.0)	11.0(9.0–12.0)	＜0.001

Individuals who developed AKI had higher LAR levels compared to those who did not. Based on this observation, we further assessed the association between LAR levels and the risk of AKI in post-cardiac surgery patients through logistic regression analysis. Univariate logistic analysis revealed that the LAR index was a significant predictor of AKI in post-surgery patients (Model 1: OR 2.150, P < 0.001). However, after adjusting for confounding factors, multivariate logistic analysis showed that the LAR index was not an independent predictor of AKI (Model 3: OR 1.405, P = 0.064, Table 2).

**Table 2:** Logistic regression analyses regarding the association between the LAR index and the occurrence of AKI in surgical populations.

LAR index	OR	95 %CI	P valve
Model 1	2.150	1.540–3.040	＜0.001
Model 2	1.916	1.362–2.722	0.002
Model 3	1.405	0.985–2.024	0.064

LAR index, lactate to albumin ratio index;OR, odds ratio; CI, confidence interval.

Model 1: unadjusted.

Model 2: Adjusted for age, gender, DBP,MAP and SpO2.

Model 3: Adjusted for age, gender, DBP, MAP, creatine, sodium, potassium, calcium, Hb, SpO2, SOFA, BUN, FBG, ALT,AG, CHF, CKD, T2DM, COPD.

Overall, although the LAR levels were higher in patients undergoing cardiac surgery who developed acute kidney injury, LAR cannot be regarded as an independent risk factor for predicting the occurrence of acute kidney injury.

### 3.2 Baseline characteristics and clinical outcomes of patients with AKI after cardiac surgery

Among the 5,965 patients who developed AKI, 30 died during hospitalization, and 29 died during their ICU stay. As shown in Tables S2 and S3, LAR levels were strongly higher in non-survivors compared to survivors, both during hospitalization and ICU stays (P < 0.001).

**Fig. 2:**
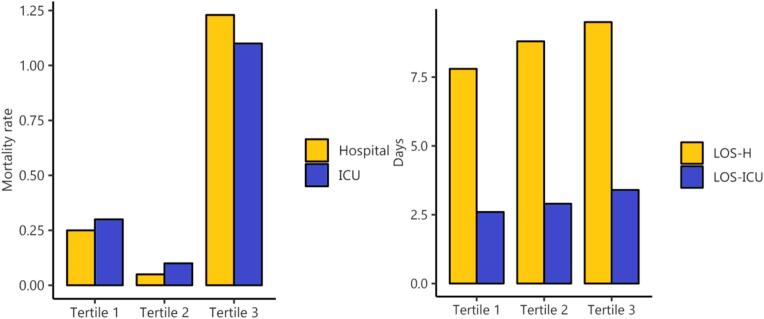
Endpoints stratified by tertiles of LAR index in the patients with AKI after cardiac surgery.

We divided patients into three groups based on LAR tertiles, revealing baseline characteristic differences between the groups (Table 3). As shown in Table 4, patients with higher LAR levels had strongly higher in-hospital and ICU mortality rates, as well as longer LOS-H and LOS-ICU, compared to those with lower LAR levels (P < 0.001). Fig. 2 further illustrates a rising trend in LOS-H and LOS-ICU with increasing LAR tertiles. Although this trend was not observed for in-hospital and ICU mortality rates, we did find that mortality rates in Tertile 3 were substantially higher than those in Tertiles 1 and 2, with in-hospital mortality notably exceeding ICU mortality. The SOFA score serves as a core tool in critical care medicine for quantitatively assessing the severity of organ dysfunction/failure in critically ill patients. We found that the combination of LAR and SOFA score enhances the screening value for mortality in patients with AKI (Table S5).

**Table 3:** Baseline characteristics of patients with AKI after cardiac surgery stratified based on the tertiles of the LAR index.

Characteristics	Tertile 1 (n = 1980)	Tertile 2 (n = 2027)	Tertile 3 (n = 1958)	P valve
Demographic data				
Age, y	69.0(61.0–77.0)	69.0(62.0–77.0)	70.0(62.0–77.0)	0.546
Gender, n(%)				0.068
Female	619(31.3)	566(27.9)	578(29.5)	
Male	1361(68.7)	1461(72.1)	1380(70.5)	
SBP, mmHg	113.0(102.7–125.0)	112.0(103.0–124.0)	112.0(101.0–123.0)	0.051
DBP, mmHg	58.0(52.0–65.0)	58.0(52.0–66.0)	58.0(52.0–65.0)	0.588
MAP, mmHg	77.0(769.0–85.0)	77.0(69.0–85.0)	77.0(69.0–85.0)	0.206
HR, bpm	80.0(74.0–86.0)	80.0(73.0–86.0)	80.0(74.0–86.0)	0.338
RR, bpm	14.0(12.0–16.0)	15.0(12.0–17.0)	15.0(12.0–17.0)	0.003
SpO2, %	100.0(100.0–100.0)	100.0(99.0–100.0)	100.0(99.0–100.0)	＜0.001
SOFA	5.0(3.0–7.0)	5.0(3.0–7.0)	5.0(4.0–7.0)	＜0.001

Comorbidities, n(%)				
Hypertension	1187(59.9)	1240(61.174)	1196(61.083)	0.679
T2DM	552(27.9)	668(33.0)	750(38.3)	＜0.001
CHF	439(22.2)	512(25.3)	518(26.5)	0.006
Stroke	151(7.7)	162(8.0)	150(7.7)	0.892
COPD	198(10.0)	203(10.0)	212(10.8)	0.619
CKD	237(12.0)	254(12.5)	237(12.1)	0.851
IHD	1428(72.1)	1546(76.3)	1512(77.2)	＜0.001

Laboratory data				
WBC, M/mcl	6.8(5.5–8.2)	7.1(5.9–8.7)	7.4(6.2–9.1)	＜0.001
PLT, K/mcl	140.0(112.0–177.0)	140.0(113.0–174.0)	145.0(115.0–180.0)	0.059
Hb, g/dL	9.4(8.2–10.7)	9.5(8.4–10.7)	9.6(8.3–10.8)	0.037
AST	22.0(18.0–28.0)	22.0(18.0–29.0)	23.0(18.0–30.0)	0.003
ALT	19.0(14.0–26.0)	19.0(14.0–28.0)	21.0(15.0–30.0)	＜0.001
TBIL	0.5(0.4–0.7)	0.5(0.4–0.7)	0.5(0.4–0.7)	0.558
LAC	0.9(0.8–1.0)	1.3(1.2–1.5)	1.8(1.6–2.1)	＜0.001
ALB	4.1(3.8–4.4)	3.9(3.6,4.3)	3.8(3.2–4.1)	＜0.001
FBG	98.0(88.0–111.0)	101.0(90.0–115.0)	104.0(93.0–120.0)	＜0.001
HbA1c	5.7(5.4–6.2)	5.9(5.5–6.5)	6.0(5.6–6.8)	＜0.001
BUN	15.0(12.0–19.0)	15.0(12.0–18.0)	15.0(12.0–18.0)	0.827
Creatinine	0.8(0.7–1.0)	0.8(0.7–1.0)	0.8(0.7–1.0)	0.473
Sodium	138.0(136.0–139.0)	137.0(136.0–139.0)	138.0(136.0–139.0)	0.097
Potassium	4.0(3.7–4.2)	4.0(3.8–4.2)	4.0(3.7–4.3)	0.580
Calcium	8.3(8.0–8.6)	8.3(8.0–8.7)	8.3(8.0–8.6)	0.573
AG	10.0(9.0–12.0)	11.0(9.0–13.0)	11.0(10.0–13.0)	＜0.001

**Table 4:** Clinical outcomes of patients with AKI after cardiac surgery stratified based on the tertiles of the LAR index.

Outcomes	Tertile 1	Tertile 2	Tertile 3	P value
Primary outcomes, n(%)				
In-hospital mortality	5(0.3)	1(0.1)	24(1.2)	<0.001
ICU mortality	6(0.3)	2(0.1)	21(1.1)	<0.001

Secondary outcomes, days				
LOS-H	6.7(5.2–8.9)	7.0(5.3–9.9)	7.6(5.6–10.9)	<0.001
LOS-ICU	2.1(1.3–3.1)	2.1(1.3–3.3)	2.2(1.3–3.5)	<0.001

Overall, these findings suggest that elevated LAR may be associated with worse outcomes in patients who develop AKI following cardiac surgery.

### 3.3 Elevated LAR associated with increased mortality in Post-Cardiac surgery AKI patients

To further evaluate the association between LAR levels and survival outcomes, we performed logistic regression analysis (Table 5). Univariate logistic regression showed a significant correlation between mortality and the LAR index. For each unit increase in LAR, the odds ratio (OR) for in-hospital mortality was 5.76, and the OR for ICU mortality was 5.38. Compared to those in the lowest tertile, individuals in the highest tertile had a 4.90-fold higher risk of in-hospital mortality and a 3.57-fold higher risk of ICU mortality. After adjusting for confounding factors, this association remained robust. In the fully adjusted Model 3, each unit increase in LAR was associated with an OR of 3.64 for in-hospital mortality and 3.17 for ICU mortality. Additionally, those in the highest tertile had a 4.53-fold higher risk of in-hospital mortality and a 3.34-fold higher risk of ICU mortality compared to the lowest tertile. There was a clear trend of increasing mortality risk with rising LAR tertiles (P for trend <0.05). Furthermore, pearson correlation analysis demonstrated a significant relationship between LAR and factors such as LOS-H, LOS-ICU, and SOFA scores (Table S4).

**Table 5:** Logistic regression analyses for the association between the LAR index and mortality in patients with AKI after cardiac surgery.

Variables	**Model 1**		**Model 2**		**Model 3**	
	OR(95 %CI)	P value	OR(95 %CI)	P value	OR(95 %CI)	P value
**In-hospital mortality**						
Per 1 Unit increase	5.76(2.68–11.40)	＜0.001	3.66(1.33–8.89)	0.008	3.64(1.24–9.68)	0.013
Tertile 1	1 reference		1 reference		1 reference	
Tertile 2	0.20(0.01–1.20)	0.140	0.19(0.01–1.21)	0.135	0.19(0.01–6.23)	0.136
Tertile 3	4.90(2.03–14.58)	0.001	4.23(1.73–12.67)	0.004	4.53(1.74–14.31)	0.004
P for trend	＜0.001		＜0.001		＜0.001	

**ICU mortality**						
Per 1 Unit increase	5.38(2.41–10.70)	＜0.001	3.46(1.22–8.61)	0.013	3.17(1.01–8.78)	0.013
Tertile 1	1 reference		1 reference		1 reference	
Tertile 2	0.32(0.05–1.41)	0.170	0.32(0.05–1.40)	0.170	0.19(0.01–1.23)	0.181
Tertile 3	3.57(1.52–9.74)	0.006	3.10(1.31–8.52)	0.016	3.34(1.32–9.76)	0.016
P for trend	0.001		0.004		0.004	

Model 1: unadjusted.

Model 2: Adjusted for age, gender, HR and SpO2.

Model 3: Adjusted for age, gender, HR, SpO2, SOFA, FBP, HbA1C, creatine, sodium, potassium, AG, Hb, PLT, BUN, CHF, CKD, T2DM, COPD, Hypertension, Stroke.

Overall, these findings suggest that elevated LAR is strongly associated with poor prognosis in post-surgery AKI patients, particularly in terms of in-hospital and ICU mortality.

### 3.4 Restricted cubic spline analysis

**Fig. 3:**
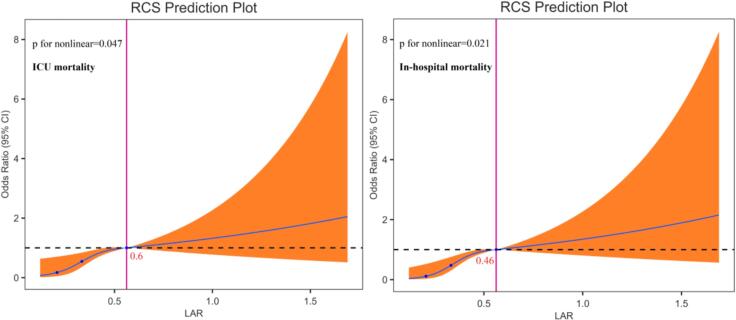
RCS curves of the LAR index in relation to mortality among patients with AKI after cardiac surgery.

To further illustrate the relationship between LAR and adverse outcomes, we employed restricted cubic spline (RCS) analysis (Fig. 3). After adjusting for covariates in Model 3, we identified a nonlinear correlation between LAR and both in-hospital and ICU mortality. Threshold effect analysis revealed a turning point for ICU mortality at an LAR value of 0.6 and for in-hospital mortality at 0.46. The findings indicate that when LAR is below these thresholds, mortality risk remains relatively low, but once LAR exceeds these points, the risk increases sharply.

### 3.5 Subgroup analysis

**Fig. 4:**
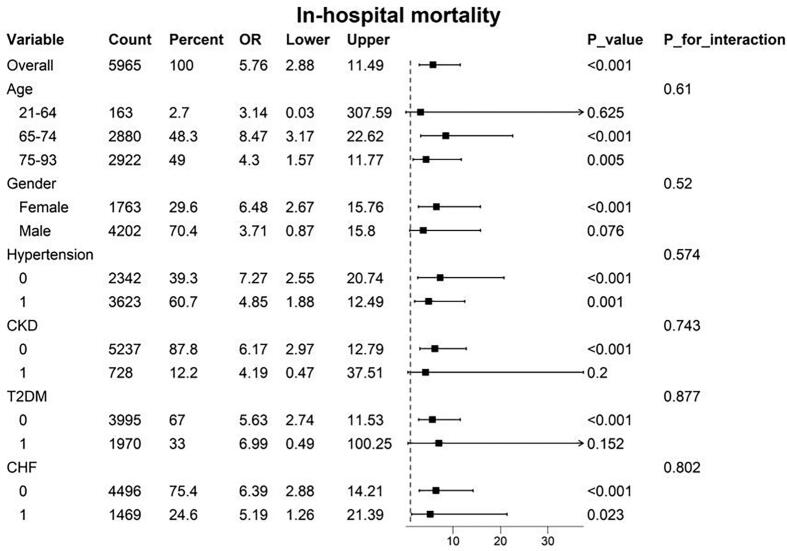
Subgroup analyses for the association between the LAR index and in-hospital mortality.

**Fig. 5:**
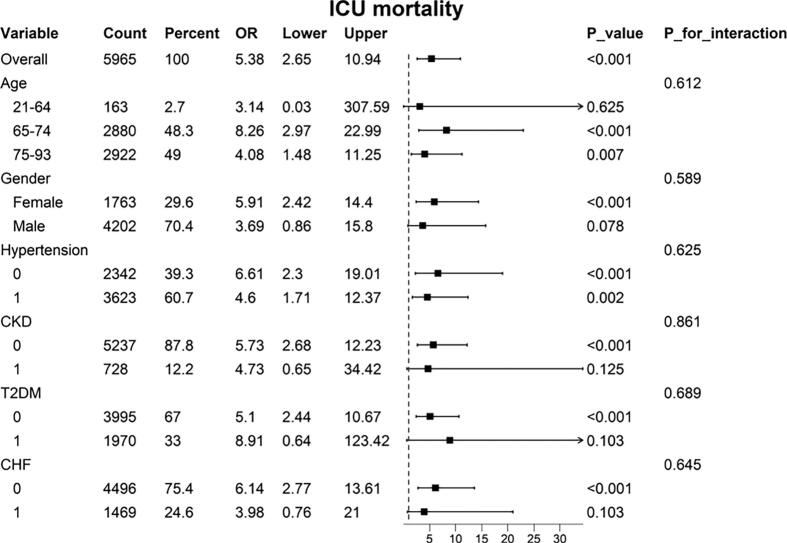
Subgroup analyses for the association between the LAR index and ICU mortality.

To assess the robustness of the association between LAR and in-hospital and ICU mortality across different subgroups, we further estimated the predictive role of LAR on mortality outcomes (Fig. 4, Fig. 5. The results consistently showed that elevated LAR was associated with increased in-hospital and ICU mortality in elderly patients, females, and individuals without chronic kidney disease, diabetes, or chronic heart failure. Notably, a strong correlation between LAR and in-hospital mortality was observed in patients with chronic heart failure. However, no significant interactions were found across subgroups defined by age, gender, hypertension, diabetes, chronic kidney disease, or chronic heart failure (P > 0.05).Furthermore, we found that for individual patients who underwent valve surgery, a higher LAR was associated with an increase in in-hospital and ICU mortality (Table S6-S7).

## 4 Discussion

We conducted a large retrospective cohort study using data from 11,624 post-cardiac surgery patients in the MIMIC-IV database and found that patients with postoperative AKI showed greater LAR elevation than those without AKI, but LAR was not an independent AKI predictor in multivariate analysis. Even after adjusting for potential confounders, elevated LAR levels were closely associated with increased risks of in-hospital and ICU mortality in post-cardiac surgery AKI patients. This is the first study to explore the relationship between LAR and adverse outcomes in this population, highlighting its previously unrecognized importance.

Each year, over 2 million cardiac surgeries are performed globally [11], with cardiac surgery-associated acute kidney injury (CSA-AKI) occurring in up to 40 % of cases, making it the second leading cause of AKI in the ICU. Perioperative mortality for severe cardiac surgery-associated acute kidney injury is 3 to 8 times higher [12], and around 3 % of patients require temporary renal replacement therapy (RRT) [13]. Even patients who fully recover kidney function remain at elevated risk for chronic kidney disease (CKD) and mortality [14,15].The mechanisms behind CSA-AKI are not fully understood but are believed to involve nephrotoxic medications [[16], [17], [18], [19]], excessive preoperative diuresis, intraoperative hemofiltration [11], inflammatory responses due to blood component interactions [20], and ischemia–reperfusion injury. These factors collectively exacerbate renal dysfunction throughout the perioperative period. Perioperative prevention of AKI in cardiac surgery relies on integrated strategies: hemodynamic optimization via continuous blood pressure monitoring with targeted renoprotective agents (e.g., levosimendan for antioxidant/anti-apoptotic effects) [[21], [22], [23]]; precise fluid management maintaining euvolemia to prevent endothelial injury from volume extremes [24,25]; prompt vasopressor support (norepinephrine/vasopressin) restoring renal perfusion during hypotension [26,27]; and continuous renal replacement therapy (CRRT) for refractory electrolyte/acid-base disturbances [28,29]. However, the absence of patient treatment data in this study may introduce confounding in the observed association between LAR and adverse outcomes, potentially obscuring the specific mechanisms through which LAR contributes to acute renal failure. Future investigations should incorporate perioperative therapeutic variables—such as those described above—to control for treatment-related confounding and elucidate relationships between LAR, acute renal failure, and clinical outcomes across varying clinical approaches.

With no specific pharmacological interventions to prevent CSA-AKI, early detection of subtle renal changes (subclinical AKI) is crucial. Biomarkers such as cystatin C (CysC), neutrophil gelatinase-associated lipocalin (NGAL), and kidney injury molecule-1 (KIM-1) can detect renal impairment before increases in serum creatinine. The lactate-to-albumin ratio (LAR) has emerged as a significant indicator for predicting poor outcomes across various critical illnesses. Elevated lactate levels reflect tissue hypoperfusion [30], while albumin is essential for maintaining plasma osmotic pressure [31] and correlates with disease prognosis [32]. However, both biomarkers are influenced by multiple factors [33], limiting their predictive accuracy when used in isolation. Integrating these indicators, studies have found that LAR serves as an independent predictor of all-cause mortality within 28 days for acute pancreatitis patients, outperforming the SOFA score [34]. In acute respiratory distress syndrome (ARDS), LAR correlates positively with 28-day mortality, exceeding predictions based on lactate or albumin alone and comparable to the SPASII score [35]. LAR has also been shown to be a poor prognostic indicator of all-cause mortality in patients with sepsis, whether admitted in the emergency department or on the ward, and correlates with the severity of sepsis [5,36].LAR is also linked to other conditions, including burns and traumatic brain injury [37,38]. A recent study found that LAR correlates with both short-term and long-term mortality in critically ill patients with cardiac surgery-associated acute kidney injury [39], aligning with our results.

However, no studies have yet investigated the relationship between LAR and adverse outcomes in patients with CSA-AKI. Our subgroup analysis revealed that, in addition to older adults and females, elevated LAR was associated with increased in-hospital and ICU mortality in patients without comorbidities. This association may stem from patients' nutritional status, chronic inflammation, or interactions with perioperative factors. A *meta*-analysis indicated that cardiac surgery-associated acute kidney injury incidence is lower in coronary artery bypass grafting (CABG) patients (19 %) compared to those undergoing valve surgery (27.5 %) or aortic surgery (29.0 %).This study showed no statistically strong difference among the three surgeries in terms of the occurrence of acute kidney injury. However, subgroup analysis indicated that a higher LAR was associated with an increased in-hospital mortality and intensive care unit mortality for individual patients undergoing valve surgery.

Our study has several limitations. Firstly, we were unable to evaluate its dynamic changes throughout the clinical course of critical illness by measuring LAR only at ICU admission,. This single-timepoint approach may miss clinically significant variations in LAR levels that could provide valuable insights into the evolving pathophysiology of postoperative AKI. Further continuous LAR measurements will be conducted at predetermined time intervals in the future. This longitudinal assessment will enable researchers to more clearly establish the temporal correlation between LAR patterns and renal outcomes, and potentially identify clinically significant trends in LAR dynamics. Secondly, we did not include the treatment regimens of the study participants, leaving us unable to determine whether different medications could improve patient outcomes, potentially introducing bias in the results. This opens avenues for future research in this area.

## 5 Conclusion

The LAR index appears to be associated with the adverse outcomes of AKI following cardiac surgery. Patients who develop AKI post-cardiac surgery and have elevated LAR levels face higher in-hospital and ICU mortality rates, especially after cardiac valve surgery. Our study highlights the previously unreported role of the LAR index as an independent risk factor for mortality in cardiac surgery-related AKI. Further large-scale studies are needed to determine whether identifying or intervening in patients with elevated LAR levels can positively impact clinical outcomes in this population.

## 6 Data Availability statement

The publicly available datasets in this study can be found online. The names of the repository/repositories and accession number(s) can be found below: https://mimic-iv.mit.edu/. Further inquiries can be directed to the corresponding author.

## Ethics statement and consent to participate

The MIMIC-IV database was approved by the Massachusetts Institute of Technology (Cambridge, MA) and Beth Israel Deaconess Medical Centre (Boston, MA), and consent was obtained for the original data collection.

## Author Contributions

CW, XS and KC designed the work. CW and XS record and summarized the patient of MIMIC features. LW, SY, SC and JG analysed the rationality of whole statistical analysis. DL provided the database approval. All authors read and approved the final manuscript.

## Statement of Authorship

All authors listed above confirm their contribution to the manuscript and agree to the following statement: This author takes responsibility for all aspects of the reliability and freedom from bias of the data presented and their discussed interpretation.

CW, XS and KC designed the work. CW and XS record and summarized the patient of MIMIC features. LW, SY, SC and JG analysed the rationality of whole statistical analysis. DL provided the database approval. All authors read and approved the final manuscript.

## CRediT authorship contribution statement

**Congying Wang:** Writing – review & editing, Writing – original draft, Visualization, Methodology, Formal analysis, Conceptualization. **Xin Sun:** Formal analysis, Data curation. **Kun Chen:** Validation. **Yaqing Shi:** Supervision. **Lili Wang:** Validation, Conceptualization. **Shuxia Chen:** Validation. **Dehua Li:** Resources. **Jian Gu:** Supervision.

## Funding

This study was supported by the Medical science research project of Hebei Province (Grant No. 20200712).

## Declaration of competing interest

The authors declare that they have no known competing financial interests or personal relationships that could have appeared to influence the work reported in this paper.
