# Supplementary material for: Lactate-to-albumin ratio index correlates with the occurrence and prognosis of acute kidney injury complicated by cardiac surgery
Source: Int J Cardiol Heart Vasc. 2025 Jul 1;59:101734. doi: 10.1016/j.ijcha.2025.101734 (PMC12271795; doi:10.1016/j.ijcha.2025.101734)
Supplement: Supplementary Data 1 [file mmc1.docx]

**Supplemental Materials**

Table S1 ……………………………………………………………………………………………2

Table S2……………………………………………………………………………………………4

Table S3……………………………………………………………………………………………5

Table S4……………………………………………………………………………………………6

Table S5……………………………………………………………………………………………6

Table S6……………………………………………………………………………………………7

Table S7……………………………………………………………………………………………7

**Table S1.** Variance inflation factor between variables.

| Variables | VIF |
| --- | --- |
| Age, | 1.355 |
| Gender | 1.388 |
| SBP | 2.022 |
| DBP | 2.500 |
| MAP | 2.634 |
| HR | 1.131 |
| RR | 1.056 |
| SpO2 | 1.119 |
| SOFA | 1.293 |
| Hypertension | 1.404 |
| T2DM | 1.668 |
| CHF | 1.245 |
| Stroke | 1.024 |
| COPD | 1.038 |
| CKD | 1.497 |
| IHD | 1.225 |
| WBC | 1.144 |
| PLT | 1.304 |
| Hb | 1.418 |
| AST | 2.570 |
| ALT | 2.503 |
| TBIL | 1.117 |
| FBG | 1.234 |
| HbA1c | 1.759 |
| BUN | 2.030 |
| Creatinine | 1.866 |
| Sodium | 1.066 |
| Potassium | 1.116 |
| Calcium | 1.054 |
| AG | 1.246 |
| In-hospital death | 2.831 |
| ICU death | 2.863 |
| LOS-H | 1.715 |
| LOS-ICU | 1.676 |

**Abbreviation:**

HR (heart rate), RR (respiratory rate), SBP (systolic blood pressure), DBP (diastolic blood pressure), MAP (mean arterial pressure), SpO2 (saturation of peripheral oxygen), T2DM (type 2 diabetes) , CHF (chronic heart failure), COPD (chronic obstructive pulmonary disease), IHD (ischemic heart disease), CKD (chronic kidney disease), WBC (white blood cell count), PLT (platelet count), Hb (hemoglobin), AST (aspartate aminotransferase), ALT (alanine aminotransferase), BUN (blood urea nitrogen), FBG (fasting blood glucose), HbA1C (glycosylated hemoglobin), AG (anion gap), SOFA (sequential organ failure assessment), LOS-H (length of stay in hospital) and LOS-ICU (the length of stay in ICU)

**Table S2.** Baseline characteristics between survivors and non-survivors during hospital stay.

| Characteristics | Survivors  (n=5935) | Non-survivors (n=30) | P valve |
| --- | --- | --- | --- |
| Demographic data |  |  |  |
| Age, y | 69.0(62.0-77.0) | 74.0(59.0-84.0) | 0.134 |
| Gender, n(%) |  |  | 0.039 |
| Female | 1749(29.5) | 14(46.7) |  |
| Male | 4186(70.5) | 16(53.3) |  |
| SBP, mmHg | 112.3(102.0-124.0) | 113.0(101.0-131.0) | 0.058 |
| DBP, mmHg | 58.0(52.0-65.0) | 57.0(52.0-67.0) | 0.633 |
| MAP, mmHg | 77.0(69.0-85.0) | 78.0(69.0-89.0) | 0.667 |
| HR, bpm | 80.0(74.0-86.0) | 86.0(80.0-92.0) | ＜0.001 |
| RR, bpm | 15.0(12.0-17.0) | 17.0(12.0-20.0) | 0.065 |
| SpO2, % | 100.0(99.0-100.0) | 100.0(94.0-100.0) | 0.011 |
| SOFA | 4.0(3.0-6.0) | 5.0(3.0-7.0) | ＜0.001 |
| Comorbidities, n(%) |  |  |  |
| Hypertension | 3607(60.8) | 16(53.3) | 0.405 |
| T2DM | 1961(33.1) | 9(30.0) | 0.724 |
| CHF | 1453(24.5) | 16(53.3) | ＜0.001 |
| Stroke | 458(7.7) | 5(16.7) | 0.068 |
| COPD | 609(10.3) | 4(13.3) | 0.580 |
| CKD | 166(8.4) | 728(12.2) | ＜0.001 |
| IHD | 4466(75.2) | 20(66. 7) | 0.278 |
| Laboratory data |  |  |  |
| WBC, M/mcl | 7.1(5.8-8.7) | 8.4(6.2-10.0) | 0.075 |
| PLT, K/mcl | 142.0(113.0-177.0) | 115.0(91.0-192.0) | 0.177 |
| Hb, g/dL | 9.5(8.3-10.7) | 9.0(7.7-10.3) | 0.048 |
| AST | 22.0(18.0-29.0) | 23.0(17.0-30.0) | 0.462 |
| ALT | 20.0(14.0-28.0) | 17.0(14.0-28.0) | 0.320 |
| TBIL | 0.5(0.4-0.7) | 0.5(0.3-0.6) | 0.572 |
| LAC | 1.3(1.0-1.7) | 1.5(1.0-1.7) | 0.231 |
| ALB | 3.9(3.5-4.3) | 2.8(2.5-3.7) | ＜0.001 |
| LAR | 0.3(0.2-0.4) | 0.5(0.4-0.6) | ＜0.001 |
| FBG | 101.0(90.0-115.0) | 96.0(81.0-112.0) | 0.259 |
| HbA1c | 5.7(5.4-6.3) | 5.8(5.5-6.5) | ＜0.001 |
| BUN | 15.0(12.0-18.0) | 18.0(14.0-25.0) | 0.003 |
| Creatinine | 0.8(0.7-10.0) | 0.9(0.7-1.9) | 0.017 |
| Sodium | 138.0(136.0-139.0) | 137.0(134.0-140.0) | 0.411 |
| Potassium | 4.0(3.7-4.2) | 3.9(3.7-4.3) | 0.870 |
| Calcium | 8.3(8.0-8.6) | 8.3(7.8-9.0) | 0.901 |
| AG | 11.0(9.0-12.0) | 12.0(11.0-14.0) | ＜0.001 |

**Table S3.** Baseline characteristics between survivors and non-survivors during ICU stay.

| Characteristics | Survivors  (n=5936) | Non-survivors (n=29) | P valve |
| --- | --- | --- | --- |
| Demographic data |  |  |  |
| Age, y | 69.0(62.0-77.0) | 74.0(59.0-84.0) | 0.134 |
| Gender, n(%) |  |  | 0.071 |
| Female | 1750(29.5) | 13(44.8) |  |
| Male | 4186(70.5) | 16(53.2) |  |
| SBP, mmHg | 112.3(102.0-124.0) | 113.0(101.0-131.0) | 0.058 |
| DBP, mmHg | 58.0(52.0-65.0) | 57.0(52.0-67.0) | 0.633 |
| MAP, mmHg | 77.0(69.0-85.0) | 78.0(69.0-89.0) | 0.667 |
| HR, bpm | 80.0(74.0-86.0) | 86.0(80.0-92.0) | ＜0.001 |
| RR, bpm | 15.0(12.0-17.0) | 17.0(12.0-20.0) | 0.065 |
| SpO2, % | 100.0(99.0-100.0) | 100.0(94.0-100.0) | 0.011 |
| SOFA | 4.0(3.0-6.0) | 5.0(3.0-7.0) | ＜0.001 |
| Comorbidities, n(%) |  |  |  |
| Hypertension | 3608(60.8) | 15(51.7) | 0.319 |
| T2DM | 1962(33.1) | 8(27. 6) | 0.532 |
| CHD | 1455(24.5) | 14(48.3) | 0.003 |
| Stroke | 457(7.7) | 6(20.7) | 0.009 |
| COPD | 609(10.3) | 4(13.3) | 0.580 |
| CKD | 719(12.2) | 9(31.0) | 0.002 |
| IHD | 4466(75.2) | 20(66. 7) | 0.278 |
| Laboratory data |  |  |  |
| WBC, M/mcl | 7.1(5.8-8.7) | 7.1(5.5-9.2) | 0.001 |
| PLT, K/mcl | 142.0(113.0-177.0) | 115.0(91.0-192.0) | 0.177 |
| Hb, g/dL | 9.5(8.3-10.7) | 8.6(7.7-9.5) | 0.024 |
| AST | 22.0(18.0-29.0) | 23.0(17.0-29.0) | 0.984 |
| ALT | 20.0(14.0-28.0) | 17.0(15.0-22.0) | 0.204 |
| TBIL | 0.5(0.4-0.7) | 0.5(0.3-0.6) | 0.601 |
| LAC | 1.3(1.0-1.7) | 1.4(1.0-1.7) | 0.504 |
| ALB | 3.9(3.5-4.3) | 2.8(2.6-3.3) | ＜0.001 |
| LAR | 0.3(0.3-0.4) | 0.5(0.4-0.6) | ＜0.001 |
| FBG | 101.0(90.0-115.0) | 100.0(88.0-112.0) | 0.673 |
| HbA1c | 5.8(5.5-6.5) | 5.8(5.4-6.3) | 0.434 |
| BUN | 15.0(12.0-18.0) | 19.0(15.0-25.0) | ＜0.001 |
| Creatinine | 0.8(0.7-1.0) | 1.0(0.8-1.7) | 0.001 |
| Sodium | 138.0(136.0-139.0) | 138.0(134.0-140.0) | 0.609 |
| Potassium | 4.0(3.7-4.2) | 3.9(3.8-4.3) | 0.790 |
| Calcium | 8.3(8.0-8.6) | 8.3(7.8-9.0) | 0.856 |
| AG | 11.0(9.0-12.0) | 12.0(11.0-14.0) | ＜0.001 |

**Table S4.** Association between the LAR index and certain continuous variables.

| Variables | Spearman analysis | |
| --- | --- | --- |
|  | Coef | P value |
| LOS-H | 0.118 | ＜0.001 |
| LOS-ICU | 0.097 | ＜0.001 |
| SOFA | 0.064 | ＜0.001 |
| Creatine | 0.028 | 0.014 |

**Table S5.** Clinical outcomes of patients with AKI after cardiac surgery stratified based on the tertiles of the LAR index with the SOFA score.

| Outcomes | Tertile 1 | Tertile 2 | Tertile 3 | P value |
| --- | --- | --- | --- | --- |
| Primary outcomes, n(%) | | | | |
| In-hospital mortality | 2(0.09) | 1(0.05) | 27(1.3) | <0.001 |
| ICU mortality | 1(0.05) | 2(0.1) | 26(1.3) | <0.001 |
| Secondary outcomes, days | | | | |
| LOS-H | 6.6(5.1-8.9) | 6.7(5.2-9.2) | 8.0(5.9-11.5) | <0.001 |
| LOS-ICU | 1.9(1.3-3.0) | 2.0(1.3-3.1) | 2.4(1.4-4.2) | <0.001 |

**Table S6.** Subgroup analysis of the association between LAR index and in-hospital mortality for different types of surgeries.

| Variable | n | OR | 95%CI | P value |
| --- | --- | --- | --- | --- |
| patients | 4890 | 6.039 | (2.92,12,50) | 0.000 |
| Surgery | | | | |
| 1 | 2938 | 8.252 | (0.20,343.41) | 0.267 |
| 2 | 1713 | 4.980 | (1.27,19.53) | 0.021 |
| 3 | 239 | 2.506 | (0.98,6.39) | 0.054 |

Note:Surgery1:CABG; Surgery2:valvular surgery; Surgery3:aortic surgery

**Table S7.** Subgroup analysis of the association between LAR index and in ICU mortality for different types of surgeries.

| Variable | n | OR | 95%CI | P value |
| --- | --- | --- | --- | --- |
| patients | 4890 | 5.980 | (2.82,12,70) | 0.000 |
| Surgery | | | | |
| 1 | 2938 | 0.168 | (0.00,257.60) | 0.267 |
| 2 | 1713 | 5.889 | (1.36,25.52) | 0.018 |
| 3 | 239 | 2.512 | (0.99,6.40) | 0.054 |

Note:Surgery1:CABG; Surgery2:valvular surgery; Surgery3:aortic surgery
